# Experience with precision genomics and tumor board, indicates frequent target identification, but barriers to delivery

**DOI:** 10.18632/oncotarget.16057

**Published:** 2017-03-09

**Authors:** Alan H. Bryce, Jan B. Egan, Mitesh J. Borad, A. Keith Stewart, Grzegorz S. Nowakowski, Asher Chanan-Khan, Mrinal M. Patnaik, Stephen M. Ansell, Michaela S. Banck, Steven I. Robinson, Aaron S. Mansfield, Eric W. Klee, Gavin R. Oliver, Jennifer B. McCormick, Norine E. Huneke, Colleen M. Tagtow, Robert B. Jenkins, Kandelaria M. Rumilla, Sarah E. Kerr, Jean-Pierre A. Kocher, Scott A. Beck, Martin E. Fernandez-Zapico, Gianrico Farrugia, Konstantinos N. Lazaridis, Robert R. McWilliams

**Affiliations:** ^1^ Hematology/Oncology, Mayo Clinic, Phoenix, AZ, U.S.A; ^2^ Mayo Clinic Cancer Center, Phoenix, AZ, U.S.A; ^3^ Center for Individualized Medicine, Mayo Clinic, Rochester, MN, U.S.A; ^4^ Hematology, Mayo Clinic, Rochester, MN, U.S.A; ^5^ Hematology/Oncology, Mayo Clinic, Jacksonville, FL, U.S.A; ^6^ Medical Oncology, Mayo Clinic, Rochester, MN, U.S.A; ^7^ Health Sciences Research, Mayo Clinic, Rochester, MN, U.S.A; ^8^ Laboratory Medicine and Pathology, Mayo Clinic, Rochester, MN, U.S.A; ^9^ Anatomic Pathology, Mayo Clinic, Rochester, MN, U.S.A; ^10^ Schulze Center for Novel Therapeutics, Division of Oncology Research, Medical Oncology, Mayo Clinic, Rochester, MN, U.S.A; ^11^ Gastroenterology, Mayo Clinic, Rochester, MN, U.S.A; ^12^ Mayo Clinic Cancer Center, Rochester, MN, U.S.A

**Keywords:** precision medicine, cancer genomics, targeted therapeutics, genomic tumor board

## Abstract

**Background:**

The ability to analyze the genomics of malignancies has opened up new possibilities for off-label targeted therapy in cancers that are refractory to standard therapy. At Mayo Clinic these efforts are organized through the Center for Individualized Medicine (CIM).

**Results:**

Prior to GTB, datasets were analyzed and integrated by a team of bioinformaticians and cancer biologists. Therapeutically actionable mutations were identified in 65% (92/141) of the patients tested with 32% (29/92) receiving genomically targeted therapy with FDA approved drugs or in an independent clinical trial with 45% (13/29) responding. Standard of care (SOC) options were continued by 15% (14/92) of patients tested before exhausting SOC options, with 71% (10/14) responding to treatment. Over 35% (34/92) of patients with actionable targets were not treated with 65% (22/34) choosing comfort measures or passing away.

**Materials and Methods:**

Patients (*N* = 165) were referred to the CIM Clinic between October 2012 and December 2015. All patients received clinical genomic panel testing with selected subsets receiving array comparative genomic hybridization and clinical whole exome sequencing to complement and validate panel findings. A genomic tumor board (GTB) reviewed results and, when possible, developed treatment recommendations.

**Conclusions:**

Treatment decisions driven by tumor genomic analysis can lead to significant clinical benefit in a minority of patients. The success of genomically driven therapy depends both on access to drugs and robustness of bioinformatics analysis. While novel clinical trial designs are increasing the utility of genomic testing, robust data sharing of outcomes is needed to optimize clinical benefit for all patients.

## INTRODUCTION

The dawn of precision medicine has brought with it understandable early enthusiasm for genomic testing by both patients and providers. Indeed, recommendations to the general medical community on how genomic testing can be integrated into medical practice are emerging [1]. Many oncologists have adopted cancer gene mutation panel testing and larger cancer centers across the United States have implemented multidisciplinary molecular tumor boards to facilitate the incorporation of genomic data in oncology clinical decision-making [2–8]. While treatment depends on identifying actionable therapeutic targets, the definition of what constitutes an “actionable” target varies between institutions. At a minimum “actionable” is defined by the ability to treat with a targeted agent based on clinical and pre-clinical data, but additional factors such as patient geographic access to a targeted therapy have also been utilized [2–4, 7]. Frequently identified barriers to treatment based on genomic testing include difficulty with payer reimbursement for testing, and the inability to access therapeutics for non-FDA approved uses, either through insurance or clinical trials [3–7, 9].

Survey of oncology providers indicates that while interest in genomic testing is high, there is a gap in provider level of comfort and knowledge when interpreting testing results [10]. In order to address this knowledge gap, Mayo Clinic established a Center for Individualized Medicine (CIM) in collaboration with the Department of Hematology/Oncology. This collaboration yielded a multi-campus Genomic Tumor Board (GTB) to enable the interdisciplinary exchange of knowledge regarding genomic testing results of advanced cancers, as well as to investigate the utility of genomic testing in clinical practice. Here we discuss initial results from these efforts.

## RESULTS

**Figure 1:**
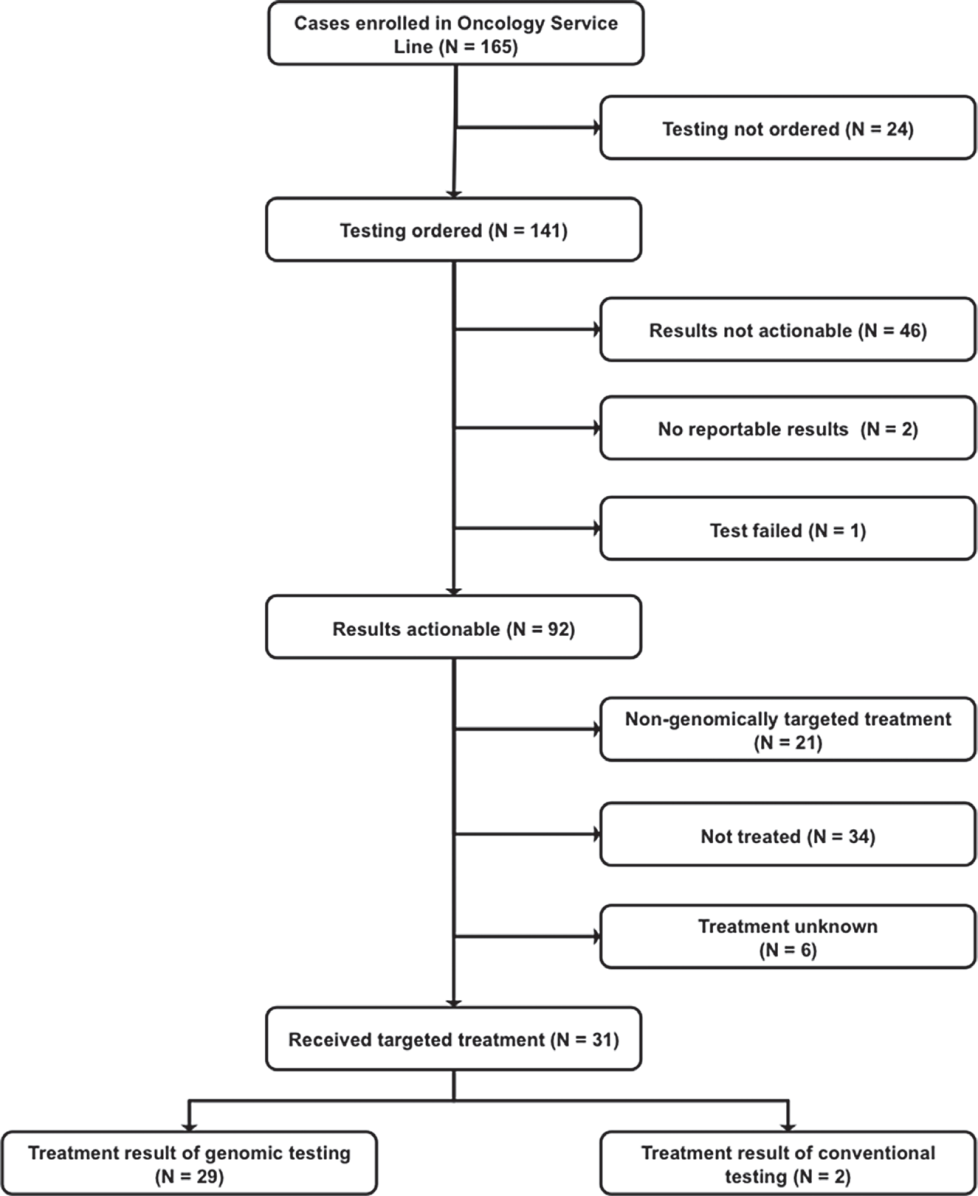
CONSORT Diagram

**Figure 2:**
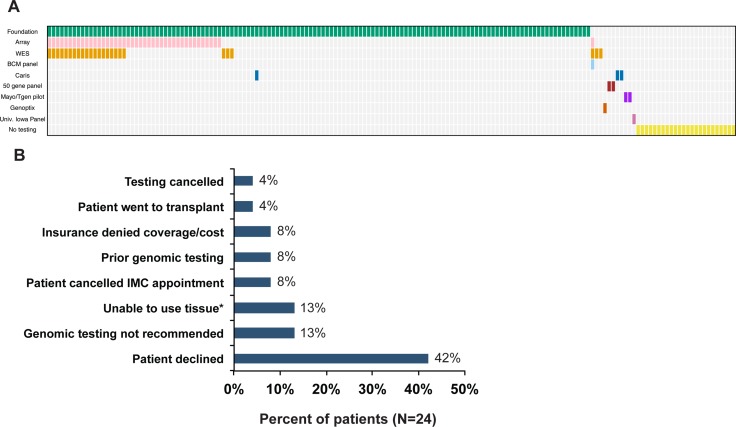
Testing Ordered (**A**) Summary of tests ordered. (**B**) Reasons testing not completed (*N* = 24). IMC = Individualizing Medicine Clinic, *Insufficient tumor, no viable tissue or tissue unavailable.

Participants with hematological malignancies and solid tumors (Table 1) were referred to the GTB between October 2012 and December 2015. Of the 165 patients referred to the GTB, 141 went on to have genomic testing ordered (Figures 1 and 2A). A single patient had no return of results due to test failure. The reasons for not proceeding with testing were varied with 42% due to the patient declining testing after consulting with the CIM physician and 8% due to cost (Figure 2B). Although studies have demonstrated improved response rates in patients with molecular aberrations matched appropriately to therapy versus patients that were not matched [8, 11], the ∼$5000 cost of genomic sequencing potentially influences patient testing choices.

**Table 1:** Patient demographics

Age range (median)	1.5 – 86 (53)
Gender male, % (*n*)	53% (88)
**Tumor type (*n* = 165)**	
**Solid (*n* = 95)**	
Gynecologic	21 (22%)
Gastrointestinal	15 (16%)
Breast	13 (14%)
Pancreas/biliary	13 (14%)
Brain	8 (8%)
Renal	4 (4%)
Lung/Thoracic	4 (4%)
Sarcoma	4 (4%)
Carcinoma unknown primary	3 (3%)
Hepatic	3 (3%)
Urothelial/bladder	3 (3%)
Adrenal	2 (2%)
Head/neck	1 (1%)
Prostate	1 (1%)
**Hematological (*n* = 70)**	
Acute Leukemia	25 (36%)
Lymphoma	24 (34%)
Chronic myeloproliferative/myelodysplastic neoplasms	15 (21%)
Myeloma	6 (9%)

Patients ranged in age from 18 months to 86 years with a median of 53 years (Table 1). Cases were divided between solid tumors (58%) and hematological malignancies (42%). The most common solid tumors presenting were gynecologic and gastrointestinal while acute leukemia and lymphoma were the most common hematological malignancies.

Prior to the GTB review of genomic testing results, an analysis team consisting of bioinformaticians and cancer biologists evaluated and annotated the findings to highlight potentially actionable targets. An aberration with known functional significance that could be therapeutically targeted with FDA approved drug(s) or clinical trial was defined as “actionable”. This contrasts with the definition of actionable by the reporting laboratories. For instance, 85% of the *KRAS* and *TP53* mutations reported in this cohort were deemed actionable by the testing laboratories, while the GTB deemed these same mutations merely informative. Some mutations were deemed to be informative even when not actionable. A mutation was defined as informative when it contributed to the understanding of the clinical course, such as association with disease aggressiveness or selection against certain therapies (e.g. KRAS mutations in colorectal cancer).

Actionable mutations were identified by the GTB in 92/141 patients for whom testing was ordered (Table 2, Figure 1) with 28% (39/141) of tumors tested possessing > 1 actionable mutation. Another 8% (11/141) had informative mutations only. A quarter of patients tested (25%) had no actionable or informative targets identified by the GTB. Interestingly, while results from multiple tests (whole exome sequencing (WES), panel, array) on the same tumor showed good concordance, the additional data from the WES and array rarely contributed to the identification of new actionable targets that altered the treatment decision.

**Table 2:** Summary of actionable and informative results

Testing completed	*N* (%)
Actionable	92 (65%)
Not actionable or informative	35 (25%)
Informative	11 (8%)
No reportable results	2 (1%)
Test failed	1 (< 1%)

**Figure 3:**
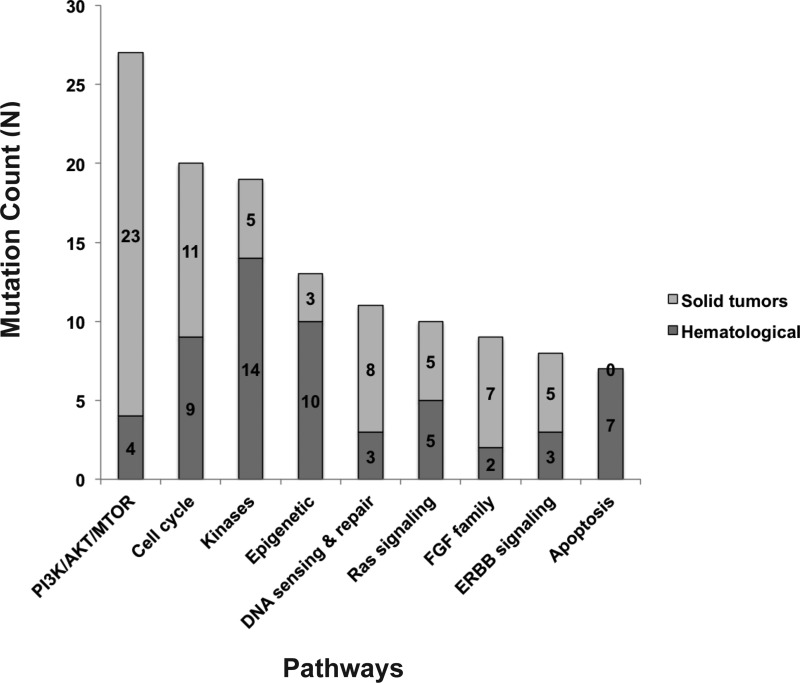
Functional pathways with therapeutically targetable actionable mutations identified by GTB

Therapeutically targetable pathways that frequently presented in this cohort include: PI3K/AKT/MTOR, cell cycle and kinases (Figure 3). Thirty-one (34%) of the patients with actionable targets went on to receive genomically targeted therapy. Treatment was based on the genomic testing results for 29 of these patients while two were based on results from conventional immunohistochemistry staining.

Treatment success was defined as all patients who derived clinical benefit from a genomically informed therapy that would not be indicated as SOC for that tumor type. The Mayo Clinic GTB experience of a Clinical Benefit Rate of 8% (13/165) on an intent to treat basis (Tables 3 and 4) is comparable to the 6% response rate expected in phase I clinical studies [12]. Some patients received testing before exhausting standard of care (SOC) options (14/92), thus elected to continue SOC treatment before targeted therapy (Table 3). The response rate among these patients was 71% (10/14). Notably, 45% (13/29) of those who pursued genomically directed targeted therapy had a clinical response, demonstrating the value of genomic testing in effectively selecting targeted therapies in patients who have exhausted SOC options or for whom few therapeutic options are available. Another 34 (37%) patients were not treated at all. Common reasons for lack of treatment were patient death/comfort measures (65%), observation (18%), and inability to access the recommended drug/insurance denial (15%). Follow up data was not available on 7% of patients.

**Table 3:** Treatment and clinical results for cases with actionable results

	Treatment result of genomic testing (N = 29)	N (%)
**Targeted therapy (*N* = 31)**	Clinical response	13 (45%)
	No clinical response	12 (41%)
	Treated elsewhere - unknown response	2 (7%)
	Passed away prior to response evaluation	1 (3%)
	Response evaluation not completed yet	1 (3%)
	**Treatment result of IHC testing (*N* = 2)**	
	Response evaluation not completed yet	1 (50%)
	Clinical response	1 (50%)
**Other treatment options (*N* = 61)**	**Received non-genomically driven treatment (*N* = 21)**	
	Continued standard of care*	14 (67%)
	Enrolled in clinical trial	3 (14%)
	Treated elsewhere	3 (14%)
	Unable to tolerate recommended drug	1 (5%)
	**Not treated (*N* = 34)**	
	Comfort measures/death near return of results	22 (65%)
	Observation	6 (18%)
	Unable to obtain drug	2 (6%)
	Insurance denied	2 (6%)
	Patient declined	1 (3%)
	Trials not local	1 (3%)
	**Unknown if treated (*N* = 6)**	

SOC = Standard of Care.

*Standard of care options not exhausted at time of genomic testing.

**Table 4:** Patients receiving genomically targeted therapy

Cancer		Actionable target	Drug	Clinical Benefit
Complete and partial responses, and stable disease	Diffuse large B cell lymphoma	CARD11 T367M	Ibrutunib & lenolidamide	CR
	Metastatic NSCLC	EGFR amp, T790M	Cetuximab & afatinib	CR
	Bladder urothelial carcinoma	ERBB2 amp	HP	CR 8 mo+
	Metastatic urethral adenocarcinoma	EGFR overexpression	Erlotinib	CR 6 mo+
	Metastatic esophageal	EGFR amp	Cetuximab & 5-fluorouracil	PR
	Cholangiocarcinoma	RBPMS-NRG1, ERRFI1 codon deletion	HP, then erlotinib	PR 6 mo, SD 3 mo
	Cholangiocarcinoma	FGFR2-TACC3 fusion	Ponatinib	SD 4 mo
	Pancreatic	ERBB3 amp	Gemcitabine & erlotinib	SD 4 mo
	Ovary granulosa cell	AKT1 W80R	Temsirolimus, Ixabepilone	SD 4 mo+
	Metastatic meningioma	BAP1 W52X	Vorinostat	SD 2 mo
	Metastatic duodenal cancer	ERBB2 A293T, S310Y, V777L	HP	SD 2 mo (deceased)
	Metastatic breast cancer	PIK3CA dup & E542K, AKT3 amp	Exemestane & everolimus	SD 1 mo
	T-cell large granular lymphocytic leukemia	STAT3 Y640F	Tofacitinib	SD x 12 mo
Progressive disease, unable to assess response	Cholangiocarcinoma	PIK3CA G364R subclonal, TSC1 N891fs*13	Everolimus	PD
	Clonal eosinophilia	IDH1 R132G, DNMT3A V716I & splice site 2597+1G>A	AG-120	PD
	Fibrolamellar hepatocellular carcinoma	FGFR4 copy number gain	Ponatinib	PD
	Lymphoma	BCL2 A2T, MYD88 L265P, PRDM1 splice site 291+2T>A, & ETV6 splice site 33+1G>A	Ibrutinib	PD
	Metastatic ampullary adenocarcinoma	BRAF K601E	Vemurafenib	PD
	Metastatic endometrial cancer	ALK rearrangement, ATM Y370X, BRCA2 C1200fs*1	REFMAL355	PD
	Metastatic ovarian cancer	SMO S33R	Vismodegib	PD
	Metastatic prostate cancer	BRAF G469A	Vemurafenib	PD
	Metastatic refractory colon cancer	ERBB2 amp	HP	PD
	Multiple myeloma	FLT4 P219T, IGH-MMSET, IGH-FGFR3	Pazopanib	PD
	Ovarian cancer	BRCA1 copy number gain & S1389fs*1, PIK3CA amp	BMN-673	PD
	Ovary Serous Carcinoma	PALB2 Y1183X	Veliparib	Mixed response/not tolerated
	T-cell acute lymphoblastic leukemia	PTEN C250fs*3, CHEK2 E351D, CDKN2A/B loss	Everolimus	PD
	Acute myelogenous leukemia	FLT3 D835V, MLL dup exons 2–10	EPZ5676	Passed away prior to response assessment
	Follicular lymphoma	IGH-BCL2 rearrangement	ABT-199	Treated elsewhere*
	Multiple myeloma	NRAS Q61H	SAR650984 clinical trial	Treated elsewhere*

NSCLC = non-small cell lung cancer, Amp = amplification, dup = duplication, HP = trastuzumab & pertuzumab, CR = complete response, SD = stable disease, PR = partial response, PD = progressive disease, *follow-up data not available.

An informal survey of 54 Mayo Clinic oncology faculty, fellows and advanced practitioners assessed the use of tumor genomics in the oncology practice and provider confidence in utilizing genomic testing (Supplementary Table 1). Over 75% had ordered genomic testing beyond that required for SOC. Only 7% reported being extremely comfortable with interpreting the results and over half indicated they are slightly or not at all comfortable with interpreting results. In our survey, two-thirds reported frequently not knowing what to do with a genomic result, with the majority seeking input from colleagues (78%) and/or conducting a literature search (50%) when uncertain.

## DISCUSSION

Driven by media coverage and public leaders, patients increasingly expect genomic analysis of their cancer as part of the therapeutic decision making process. While it is encouraging that 65% (92/141) of the tumors tested in this cohort had a potentially actionable target, only 32% (29/92) of these patients went on to receive genomically driven targeted therapy and 15% (5/34) were unable to gain access to the recommended therapy. This points to a myriad of drug access barriers that prevent optimal utilization of tumor genomic testing including cost and traditional clinical trial design.

One of these barriers is cost, both for the tumor genomic analysis as well as for the subsequent treatment. Testing reimbursement varies by insurance company with differing policies on how out-of-pocket costs to patients are handled. Reimbursement of treatment costs is also variable. Of those with an actionable target, 6% were not able to receive targeted therapy due to insurance denying payment. For the others, treatment was received as part of a clinical trial, insurance coverage or as compassionate care from the company manufacturing the targeted agent.

At the onset of our clinic, there were no tumor agnostic biomarker driven studies for most targets. This changed over the 3+ years of the GTB with a significant impact on the rate of initiation of targeted therapy. Many of the later patients receiving genomically targeted therapy in this cohort received treatment on a dedicated, biomarker driven, and tumor agnostic clinical trial, whereas some early patients were treated though a single patient IND process. The My Pathways study (NCT02091141) is a four-arm study with targeted agents for Her2 amplification or activating mutation (trastuzumab and pertuzumab), EGFR activating mutation (erlotinib), BRAF activating mutation (vemurafenib), or a hedgehog pathway activating mutation (vismodegib). This type of study design, known as a “basket” study, is necessary to rapidly test clinical significance of recurrent genetic aberrations across histologic tumor types. Another example is the NCI-MATCH study (NCT02465060) that offers access to a broad range of targeted agents based on genetic aberrations. For institutions without robust phase I programs, basket studies will likely be the most effective source of access to targeted drugs.

Basket studies notwithstanding, for genomics to optimally impact clinical practice, insights gained from successful treatment must be rapidly shared among clinicians and investigators. While the GTB facilitates sharing of knowledge within Mayo Clinic, cross-institutional databases linking genomic profiles and treatment outcomes are desperately needed given the rarity of specific aberration/tumor combinations. These data sharing challenges as well as potential solutions were recently outlined by the Global Alliance for Genomic Health [13]. The National Cancer Institute has taken a step in this direction with the formation of the Experimental Therapeutics Clinical Trials Network (ET-CTN) to facilitate integration of clinical trial and molecular data [14] and with the recent launch of the Genomic Data Commons to facilitate integration of genomic datasets [15].

Questions inevitably arise about the cost effectiveness of tumor sequencing. Savings can be realized from reduced use of ineffective therapies and avoidance of toxicities from unneeded treatments. Thus, the cost of tumor sequencing should be measured in contrast to the cost of treating with unselected therapies or enrolling in non-biomarker based clinical trials. While the 45% (13/29) response in the genomically targeted group is less than the SOC response of 71% (10/14), many patients in this cohort exhausted SOC options or presented with tumors for which chemotherapy options are scarce. Identification of actionable targets in patients such as these can enable a physician to determine if a standard of care drug, such as erlotinib in pancreatic cancer that is rarely used due to minimal survival benefit, may in fact be effective in treating the patient's tumor. Thus while the response to targeted therapy is less than SOC, the value of genomic testing to these patients who would otherwise pursue unselected therapies cannot be overlooked. It is also not known whether repeated biopsies to obtain “fresh” tissue are necessary for appropriate targeted therapy recommendations. Most mutations considered “drivers” are often not acquired, but present in a given patient's tumor from an early stage. The cost and potential morbidity of repetitive biopsies must be weighed against the likelihood of finding new or prior sub-detection targets from emerging clones of tumor. Liquid biopsies of circulating DNA may address this concern to some extent, however. Cost effective application of –omic approaches to cancer care for patients such as this will be enabled by utilization of basket studies and inter-institution knowledge sharing.

Nevertheless, fully realizing the potential of tumor genomics to benefit clinical care remains challenging as surveys by ourselves and others indicate the use of genomic testing in oncology practice, but discomfort with interpreting results. Less than half of those responding report being very confident in their genomic knowledge, ability to explain genomics to patients and make treatment recommendations from genomic results [10].

Clinicians must remain cautious when accessing targeted therapy outside FDA indications or clinical trials. Genomic testing can carry with it an aura of certainty that leads patients and providers to become overly reliant on laboratory reported results in lieu of proven treatments. Inherently this approach is limited, as the laboratory report cannot consider clinical context including prior therapies, comorbidities, concomitant medications, or tumor burden. In addition, the reports do not provide detailed mutation frequency information. Levels of evidence defining “actionable” are widely variable thus thorough review of available evidence or utilizing resources like a GTB before initiating off label, targeted therapy is ideal. By creating a multidisciplinary team engaged in patient case review, the GTB is structured to address these limitations by considering genomic testing results in addition to treatment options such as: surgery, ablation, radiation, new chemotherapy, or observation. For instance, the recommendation for a medulloblastoma patient with an activating *PIK3CA* mutation responding well to standard chemotherapy and radiation would be to continue SOC rather than receive a new, specific targeted therapy.

While the GTB provides a forum for a rich, in-depth discussion of genomic findings in the context of each patient, it also provides an educational opportunity for all GTB participants including strengths/weaknesses of sequencing tools or comparison of treatment options in other tumor types. Furthermore, this forum enables vetting of competing therapeutic options when multiple actionable targets and their associated treatment options require prioritization. This is accomplished through weighting of factors such as patient access to drug, comorbidities, and level of evidence supporting each treatment option in order to determine the most effective and least toxic treatment. In one case, after GTB review of a metastatic urothelial carcinoma presenting with an activating *PIK3CA* mutation and loss of *ATM*, the GTB priortized the *ATM* loss to pursue therapeutically. In another example, a prostate biopsy of a male with a pelvic mass revealed a pure squamous cell carcinoma (SCC) with no adenocarcinoma components present and negative staining for prostate specific antigen (PSA) and prostate specific acid phosphatase (PSAP). The differential diagnosis included SCC of the prostate versus SCC of the urethra or other primary site. Ultimately, genomic testing revealed a TMPRSS2-ERG fusion in the SCC, which is characteristic of prostate cancer. At other times the GTB may disagree with recommendations from the testing laboratory, in particular when defining mutations as actionable. Prior to the initiation of therapy, further testing may be recommended and may include clinical testing such as immunohistochemistry, or research testing such as RNA sequencing, mate-pair sequencing and *in vitro* functional studies of identified mutations. Thus, the GTB provides a translational link between clinicians and research scientists to assist with target prioritization or consideration of alternate targets. The experience of the GTB has promoted an evolution in our institutional practice, with an emerging consensus to begin genomic analysis early in the treatment course due to many driver mutations presenting early in the disease course [16]. This allows time for the acquisition of potential therapies in a clinically useful timeframe. At Mayo Clinic, we believe that consideration of genomic findings are best incorporated within the full clinical context of the patient's care, and the natural evolution of the GTB should be to incorporate its functions into traditional histology based tumor boards. This will require greater familiarity with genomic techniques and data by treating clinicians and pathologists, and the presence of cancer genomics scientists and bioinformaticians. While genome sequencing is capable of revealing volumes of precise information about a patient, it augments, but does not replace, modern cancer treatment options.

The development of a GTB serves many purposes as genomic testing becomes increasingly utilized in oncology practices. Not only does the GTB winnow out actionable therapeutic targets identified in testing, it also provides a forum for teaching and consideration of alternative treatment options in complex cases. Furthermore, it facilitates translational collaboration between physicians and scientists. GTBs will contribute to the ongoing evolution of tumor genomic-based treatment in oncology, along with innovations in clinical trial design, technological innovations in big data management, and regulatory changes promoting data.

## MATERIALS AND METHODS

### Data and sample collection

Patients were referred to the GTB [17] between October 2012 and December 2015. Clinical information was obtained from Mayo Clinic medical records. Informed consent was obtained for each patient participating in the CIM Hematology/Oncology research protocol approved by the Mayo Clinic Institutional Review Board (IRB 12-007850). Eligible patients were required to have a histological or cytological confirmation of a malignancy, a life expectancy > 3 months, be a candidate for a research biopsy or surgical procedure to obtain tissue, or have pre-existing tissue sample available from which DNA and RNA can be extracted and be able to provide informed consent. Waiver of consent and HIPAA (Health Insurance Portability and Accountability Act) was approved by the Mayo Clinic Institutional Review Board (IRB 16-000400) for record review of CIM Hematology/Oncology patients not enrolled in the research protocol. Two patients were sequenced under a Mayo Clinic – Translational Genomics Research Institute pilot study (IRB 10-006180 and 10-002879) described elsewhere [18]. Clinical follow-up data was collected and stored in REDCap (Research Electronic Data Capture) hosted at Mayo Clinic [19]. REDCap is a secure, web-based application designed to support data capture for research studies. Genomic data was stored on a secure, limited access server within the Mayo Clinic firewall.

### Genetic counseling and testing

Patients undergoing whole exome genomic testing first met with a Genetic Counselor to discuss the benefits and risks of genetic testing. Fresh frozen tissue specimens were collected during surgical resection or biopsy, and maintained at −80°C until nucleic acid extraction. A board certified pathologist evaluated a portion of each specimen that was processed through standard formalin fixation and paraffin embedding, plus touch preparations for the biopsies, to confirm the presence of tumor, degree of necrosis, percent cellularity and percent of tumor nuclei. The remaining portion was processed through standard preservation methods in paraffin. Testing that only required paraffin embedded samples was performed either on newly acquired or on archival tissue acquired clinically. For those undergoing whole exome sequencing, blood was also obtained to serve as the germline DNA source.

Clinical genetic testing of tumors including next-vgeneration sequencing panels, array comparative genomic hybridization (CGH) and whole exome sequencing (WES) was conducted in Clinical Laboratory Improvement Amendments (CLIA) certified laboratories. These laboratories included: Foundation Medicine (Cambridge, MA), Baylor College of Medicine (Houston, TX), Caris Life Sciences (Phoenix, AZ), Genoptix (Carlsbad, CA) and Mayo Clinic (Rochester, MN). Two patients were sequenced in a pilot study between the Mayo Clinic and the Translational Genomics Research Institute described elsewhere [18] wherein sequencing was conducted in a research setting, but confirmation of reported findings were confirmed in a CLIA certified laboratory. Panel testing and CGH were conducted on tumor only, while WES was conducted on both tumor and blood.

### Reporting of results

Upon receipt of findings an analysis team consisting of bioinformaticians and cancer biologists provided additional biological context utilizing publically available databases and functional prediction algorithms. Evidence from the literature of the mutations’ potential functional significance was reported in the context of the patient's tumor and clinical history.

The annotated findings were then presented to a multidisciplinary Genomic Tumor Board (GTB) consisting of: physicians, research scientists, cancer biologists, ethicists, pathologists, bioinformaticians, and genetic counselors from Mayo Clinic campuses in Minnesota, Arizona and Florida. Each case review included an overview by an oncologist of the patient's clinical oncology, family and genetic testing histories, and presentation of tumor genomic findings by a cancer biologist. The discussion involved all in attendance. At the close of each discussion, the GTB would formally conclude by consensus if findings: 1) were deemed actionable, 2) led to treatment recommendations and 3) were deemed informative. Conclusions were recorded in the RedCap database. In some cases, recommendations were made for additional clinical or research laboratory tests to confirm activation of therapeutically targetable pathways. If no additional testing was required, then recommendations from the GTB discussion were made to the treating physician. A Disease Oriented Group reviewed hematological malignancy patients for which only clinical testing was ordered, but if additional research testing was recommended, results were reviewed by the GTB upon receipt of research findings. Treatments provided to patients were FDA approved or provided as part of enrollment in an independent clinical trial.

### Provider survey

An assessment of the use of genomic analyses in oncology practice (Supplementary Table 1) was conducted among oncology providers at the Mayo Clinic in Rochester utilizing Survey Monkey (www.surveymonkey.com). These questions probed the providers use of and comfort level with, genomic testing in their oncology practice.

## SUPPLEMENTARY MATERIALS TABLE


