# Supplementary material for: Experience with precision genomics and tumor board, indicates frequent target identification, but barriers to delivery
Source: Oncotarget. 2017 Mar 9;8(16):27145–54. doi: 10.18632/oncotarget.16057 (PMC5432324; doi:10.18632/oncotarget.16057)
Supplement: Supplementary file 1 [file oncotarget-08-27145-s001.pdf]

Experience with precision genomics and tumor board, indicates frequent target identification, but barriers to delivery

Supplementary Materials

Supplementary Table 1: Assessment of Genomic Analyses Use in Oncology Practice

Question and answer choices		N (%)
**Q1:**	**Have you used genomic testing in your practice?**	**N = 54**
	Yes	42 (78%)
	No	9 (17%)
	Other (please specify)	3 (6%)
**Q2:**	**If yes, how comfortable do you feel interpreting the genomic information?**	**N = 46**
	Extremely comfortable	3 (7%)
	Moderately comfortable	13 (28%)
	Comfortable	6 (13%)
	Slightly uncomfortable	12 (26%)
	Not at all comfortable	12 (26%)
**Q3:**	**If you answered yes to question #1, have you not known what to do with a genomic result that you received?**	**N = 44**
	Commonly	30 (68%)
	Rarely	11 (25%)
	Never	3 (7%)
**Q4:**	**What do you usually do if you do not know what to do with a result?**	**N = 50**
	I ask a colleague	39 (78%)
	I do a literature search	25 (50%)
	I wing it	2 (4%)
	I follow the recommendations in the report	14 (28%)
	Other (please specify)	5 (10%)
**Q5:**	**Would an option to query a Genomic Tumor Board (with multidisciplinary expertise) for a result that you obtained be helpful?**	**N = 54**
	Yes, I would find that helpful	44 (81%)
	Not sure	8 (15%)
	No, I don't think it would be helpful	2 (4%)
**Q6:**	**What is your role?**	**N = 54**
	Rochester staff	23 (43%)
	Health System provider	8 (15%)
	Fellow	13 (24%)
	NP/PA	10 (19%)
